# Dataset of the vascular **e**-**L**earning during the **COVID**-19 pandemic (EL-COVID) survey

**DOI:** 10.1016/j.dib.2021.107442

**Published:** 2021-10-01

**Authors:** Nikolaos Patelis, Theodosios Bisdas, Zaiping Jing, Jiaxuan Feng, Matthias Trenner, Nyityasmono Tri Nugroho, Paulo Eduardo Ocke Reis, Stephane Elkouri, Alexandre Lecis, Lamisse Karam, Dirk Le Roux, Mihai Ionac, Marton Berczeli, Vincent Jongkind, Kak Khee Yeung, Athanasios Katsargyris, Efthymios Avgerinos, Demetrios Moris, Andrew Choong, Jun Jie Ng, Ivan Cvjetko, George A. Antoniou, Phillipe Ghibu, Alexei Svetlikov, Fernando Gallardo Pedrajas, Harm P. Ebben, Hubert Stepak, Andrii Chornuy, Sviatoslav Kostiv, Stefano Ancetti, Niki Tadayon, Akli Mekkar, Leonid Magnitskiy, Liliana Fidalgo-Domingos, Sean Matheiken, Eduardo Sebastian Sarutte Rosello, Arda Isik, Georgios Kirkilesis, Kyriaki Kakavia, Sotirios Georgopoulos

**Affiliations:** a 3rd Department of Vascular Surgery, Athens Medical Center, Greece; b National & Kapodistrian University of Athens, Greece; c Vascular surgery department, First affiliated hospital to Navy medical university, Shanghai, PR China; d Klinikum rechts der Isar, School of Medicine, Technical University of Munich, Germany; e Faculty of Medicine University of Indonesia - Cipto Mangunkusumo National Hospital, Indonesia; f Federal University Fluminense, Brazil; g Centre Hospitalier de l'Université de Montréal, Canada; h Centre Hospitalier de Troyes, France; i Hotel Dieu de France Hospital, Lebanon; j University of the Witwatersrand, South Africa; k University of Medicine and Pharmacy, Romania; l Semmelweis University, Hungary; m Amsterdam University Medical Center, The Netherlands; n VU Medical Center, The Netherlands; o Paracelsus Medical University, Klinikum Nurenberg, Germany; p University of Pittsburgh Medical Center, USA; q Duke University Medical Center, USA; r National University of Singapore, Singapore; s University Hospital Merkur, Croatia; t Pennine Acute Hospitals NHS Trust, UK; u University Hospital Hairmyres, UK; v Vascular & endovascular surgery Center, National Scientific-Clinical Memorial Hospital, ``Professor I.I. Mechnikov'', North-Western Medical University, The Russian Federation; w Hospital Quironsalud Málaga, Spain; x Poznan University of Medical Sciences, Poland; y Ternopil University Clinic, Ukraine; z University of Bologna, Italy; aa Shahid Beheshti University of Medical Sciences, Iran; bb CHU Titi Ouzou, Algeria; cc Pirogov City Hospital No1, The Russian Federation; dd Hospital Clínico Universitario de Valladolid, Spain; ee Bedford Hospital NHS Trust, UK; ff Universidad de la Republica, Uruguay; gg Erzincan Binali Yildirim University, Turkey

**Keywords:** e-learning, Training, Education, Distance learning, Vascular surgery, Surgery

## Abstract

This dataset supports the findings of the vascular e-Learning during the COVID-19 pandemic survey (the EL-COVID survey). The General Data Protection Regulation (GDPR) of the European Union was taken into consideration in all steps of data handling. The survey was approved by the institutional ethics committee of the Primary Investigator and an online English survey consisting of 18 questions was developed ad-hoc. A bilingual English-Mandarin version of the questionnaire was developed according to the instructions of the Chinese Medical Association in order to be used in mainland People's Republic of China. Differences between the two questionnaires were minor and did affect the process of data collection. Both questionnaires were hosted online.

The EL-COVID survey was advertised through major social media. All national and regional contributors contacted their respective colleagues through direct messaging on social media or by email. Eight national societies or groups supported the dissemination of the EL-COVID survey.

The data provided demographics information of the EL-COVID participants and an insight on the level of difficulty in accessing or citing previously attended online activities and whether participants were keen on citing these activities in their Curricula Vitae. A categorization of additional comments made by the participants are also based on the data.

The survey responses were filtered, anonymized and submitted to descriptive analysis of percentage.

## Specifications Table

**Table:** 

Subject	Surgery
Specific subject area	Vascular surgery
Type of data	Summary statistics Tables Charts
How data were acquired	Through an online survey, constructed using Google Form (online copy available at http://med-pie.com//el-covid-survey/el-covid-english.pdf) & SurveyLab (online copy available at http://med-pie.com//el-covid-survey/el-covid-chinese-english.pdf)
Data format	Filtered Analyzed Raw
Parameters for data collection	During the COVID-19 pandemic, vascular surgeons and vascular trainees were reached by social media or direct contact and were requested to complete an online questionnaire regarding vascular e-Learning.
Description of data collection	The data was collected through online questionnaires, which was disseminated to the international vascular society through social media. The questionnaires were created ad-hoc.
Data source location	Athens Medical Center, 56 Kifisias Avenue, Marousi 15125, Athens - Greece
Data accessibility	Online access to the raw data through Mendeley Data at http://dx.doi.org/10.17632/fv5ztss3yf.4 Partial data are available in this manuscript Other data are available in the related research article.
Related research article	[1]. Vascular e-Learning during the COVID-19 pandemic: the EL-COVID survey. Annals of Vascular Surgery 2021. DOI:10.1016/j.avsg.2021.08.001

## Value of the Data

• These data provide a global insight on the appreciation of the vascular e-Learning, as well on the advantages and disadvantages of current vascular e-Learning activities.
• The presented data are essential since this is the largest survey of its kind regarding the appreciation of vascular e-Learning with the exponential growth in its usage during the COVID-19 pandemic.
• All researchers on vascular surgery training and education could benefit from these data. Researchers from other relevant medical fields could also use these data to support any necessary transition from traditional education and training to e-Learning.
• Our data could be compared to or be complementary to similar data from newer studies.
• These data may assist medical schools or institutions to implement the necessary changes for a partial or full transition to e-Learning.

## 1 Data Description

The dataset provides useful information based on survey data of the EL-COVID regarding the vascular e-Learning appreciation, advantages and disadvantages during the pandemic. The demographics of the 856 participants are presented in Table 1.

**Table 1:** Demographics of participants.

	n	%
**Gender**		
Male	673	78.62
Female	181	21.02
Νon-binary/LGBTQ+	2	0.23
**Εxperience level**		
Vascular surgeon with >5yrs of experience	482	56.3
Vascular surgeon with <5yrs of experience	213	24.9
Vascular Trainees	161	18.8

**Fig. 1:**
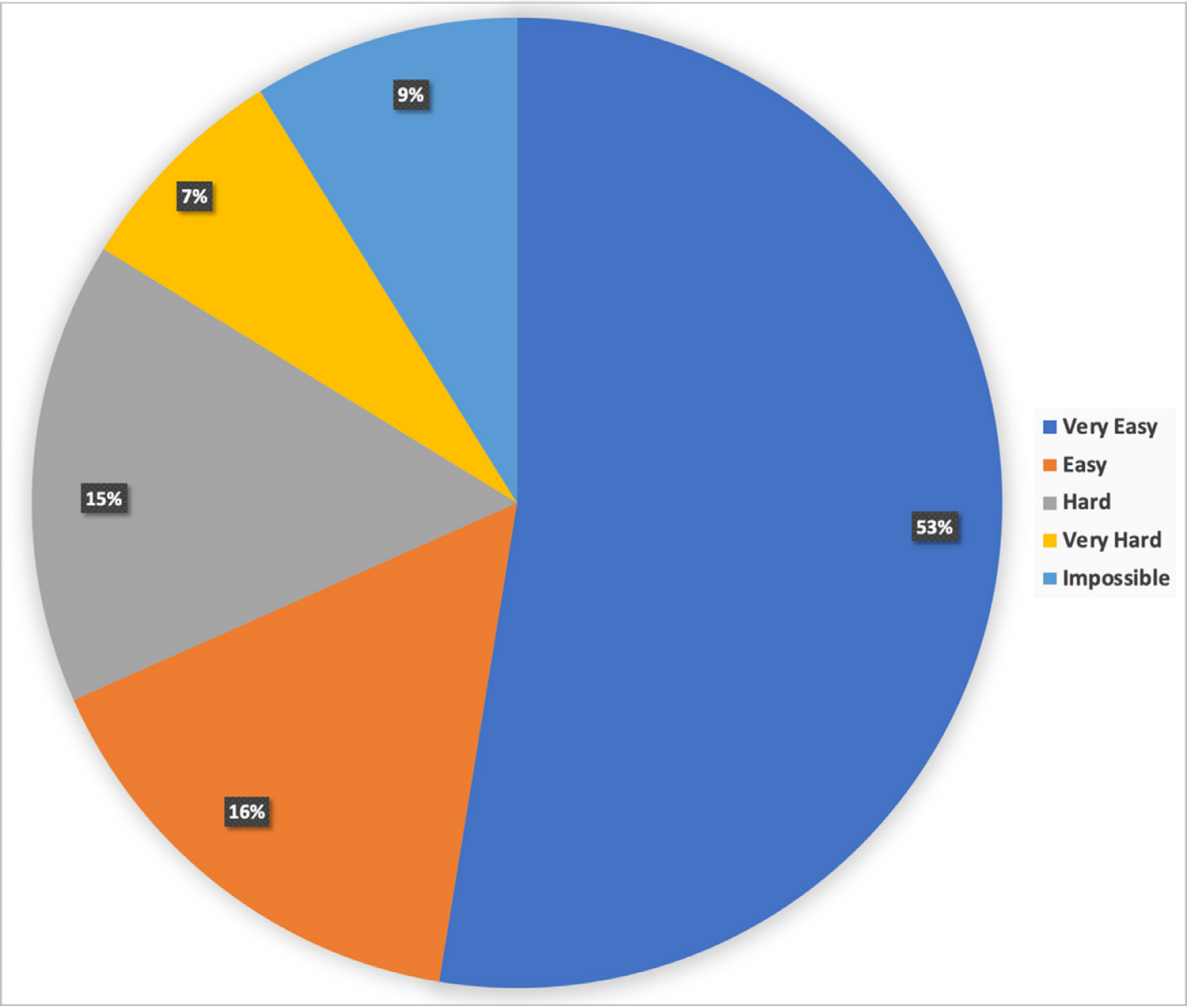
Degree of difficulty in accessing previously attended online activities.

The difficulty of accessing previously attended online training or educational activities varies from very easy to impossible. This process was considered very easy or easy in 53% and 16%, respectively. On the contrary, 7% and 9% of participants considered it very hard or impossible, respectively (Fig. 1).

**Fig. 2:**
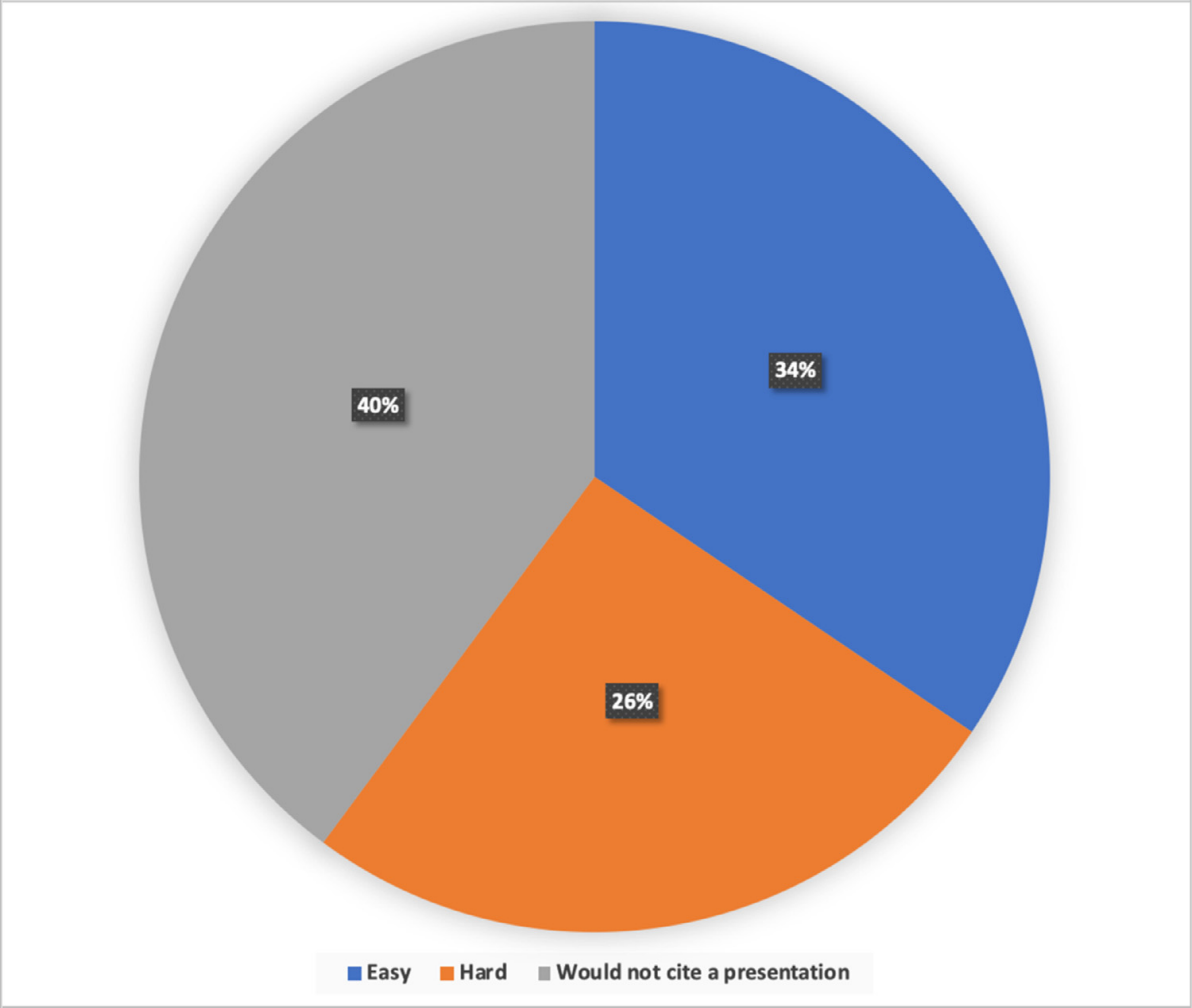
Degree of difficulty in properly citing a previously attended online activity.

The difficulty of citing a previously attended online activity varies as well. Most of the participants (40%) would not cite an online activity (Fig. 2). The rest almost equally considered citing such an activity easy or hard, in 34% and 26% of responses, respectively.

**Fig. 3:**
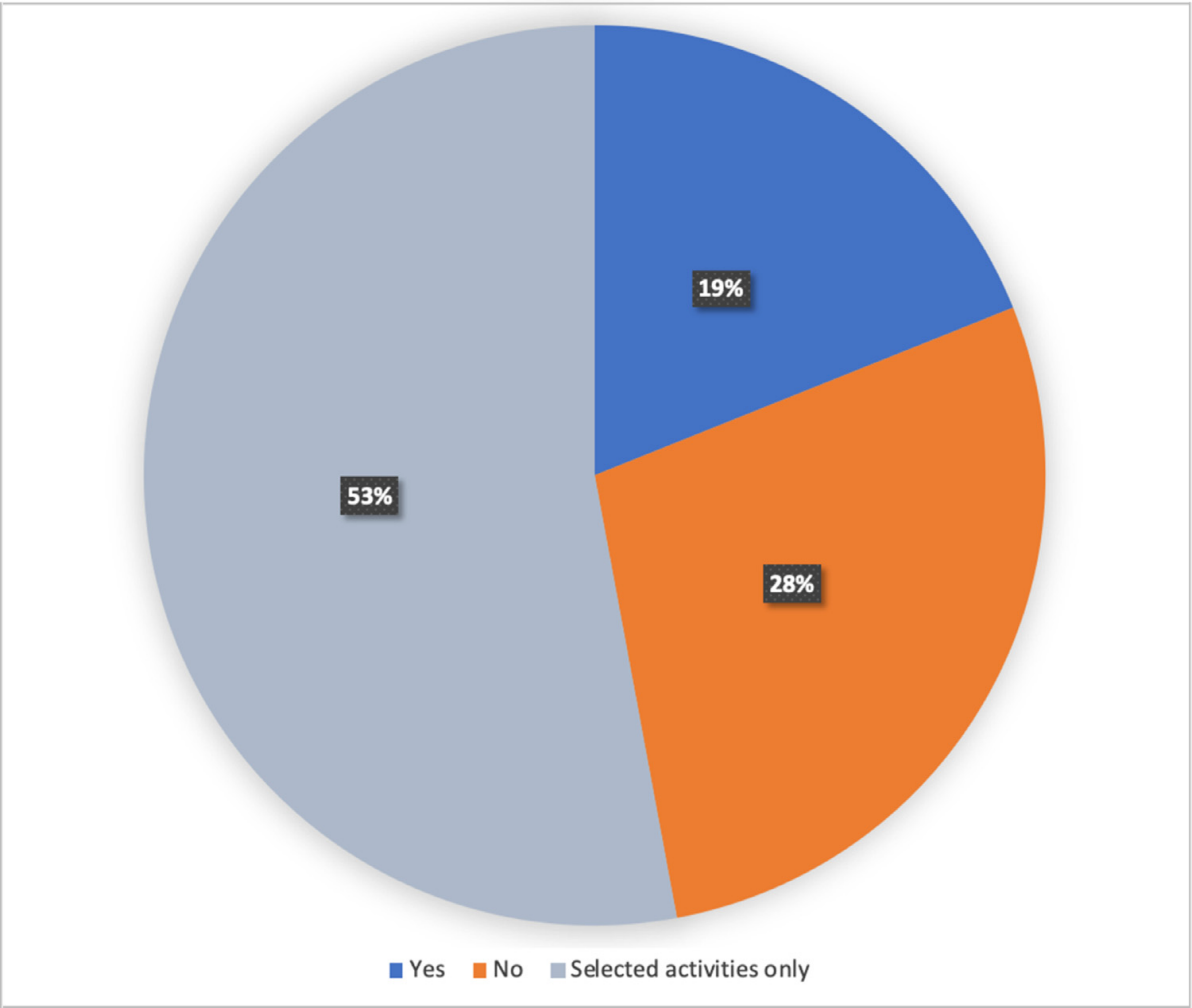
Listing of attended online activities in curricula vitae.

According to the responses to our survey, 19% of participants would list all of the online activities they have attended in their Curricula Vitae, while 24% would not list any of them. The majority (53%) would list only selected activities (e.g. those with official accreditation) (Fig. 3).

Apart from the questions of the survey, there were 328 free comments on vascular e-Learning during the pandemic. These comments were classified according to their subject and they are listed in Table 2.

**Table 2:** Comments made by the participants grouped by categories.

Comment	n
On-demand (asynchronous) content/accessibility	82
Interaction	52
Improvement of content/additional knowledge	25
Accreditation	21
Timing & length	23
Need of better organization	18
Proper advertisement/announcement	13
Time-zone consideration	12
More activities	13
Connection/technical issues	12
Audio & video improvement	9
Live cases	9
Free registration	6
Protected/allocated time	6
Copy of activity	7
More visual presentations	5
Techniques/experts' tips	5
Reference issues	4
Translation/captions	3
Evaluation/Feedback	3

**Total:**	**328**

## 2 Experimental Design, Materials and Methods

This international survey was performed using a cross-sectional method to determine the appreciation, advantages and disadvantages of vascular e-Learning during the COVID-19 pandemic.

The official language of the survey was English. The General Data Protection Regulation (GDPR) of the European Union was taken into consideration in preparing all materials of this research. The survey was approved by the ethics committee of the Primary Investigator's institution.

In our study, a vascular e-Learning activity or distance learning activity was defined as any educational or training activity that is performed exclusively online (either synchronous or asynchronous) and the main topic falls under the scope of vascular or endovascular disciplines.

The pandemic period was defined as the period between March 15th and May 15th, 2020 for Europe. For other regions, this period may slightly vary according to imposed measures by each government and epidemiological data from each region. This statement was posted in the official research page and in the two survey forms.

The design of our study required only vascular surgeons and vascular trainees to complete the survey, something that was a challenge on its own account. There are different definitions on who is a vascular surgeon or a vascular trainee. In some regions, a vascular surgeon would perform an open procedure but not an endovascular one. In other parts of the world, general surgeons with vascular training would perform some procedures, but not others. In some countries, trainees often have to go through a core surgical training program before they go ahead with vascular training, but they are not considered vascular trainees until they do the latter. So, the EL-COVID team decided not to strictly define who is a vascular surgeon, but rather used a vague definition of any surgeon who performs the majority of open and endovascular procedures. In a similar manner, a vascular trainee is any physician enrolled at any stage of an official vascular surgery training program.

An online questionnaire consisting of 18 questions was developed ad-hoc; three questions were on demographics, 14 on the e-learning experience and opinion, and one field for the participants’ email address. Filling-in the email was voluntary aiming in assisting in the verification and validation of our results. Validation of the data was initially performed by ignoring suspicious results recorded from the same computer within a small period of time and individuals who had participated more than once (based on identical IP, duplicate email address or identical demographics data within a small period of times). All data that could lead to identifying specific individuals who participated in the survey (e.g. timestamp, IP address, email address) were not shared with any parties and were deleted once data validation was complete. A single copy containing all raw data is saved by the GDPR data protection officer (NP) for future reference, if questions of data validity were risen. A bilingual English-Mandarin version of the same questionnaire was created according to the instructions of the Chinese Medical Association in order to be used in mainland People's Republic of China (PR China). Apart from the Chinese text, the only difference between the original and the bilingual questionnaires was that in the latter there was no country of practice field, as it was developed only for use within mainland PR China. Both questionnaires are available as supplementary material.

The original questionnaire was hosted on Google Forms (Mountain View CA, USA), while the bilingual version was hosted on SurveyLab (Warsaw, Poland).

The online questionnaires were available on both hosts for a period of four months. The EL-COVID survey was advertised through social media (so.me.); primarily in LinkedIn (Mountain View CA, USA) and secondarily in Twitter (San Francisco CA, USA) and Facebook (Menlo Park CA, USA). All national and regional contributors were asked to contact their respective colleagues through direct messaging on any so.me. platform or by email. Thirty-eight international and national societies were contacted in order to support the EL-COVID research by sending their members a link to the survey; with eight of them supporting our research and two declining. The above information was described in more detail in the official EL-COVID webpage hosted at med-pie.com on a non-commercial basis. All data analysis was performed using Microsoft Excel for Mac version 16.16.27.

This study has a number of limitations. Since the questionnaires were only hosted online and the survey information was disseminated through so.me., data collection might be biased against vascular surgeons and trainees who are not active in so.me. or familiar with online questionnaires. The second limitation is that it is impossible to know the exact number of vascular surgeons and trainees, since there are different vascular training curricula around the globe and vascular surgery is not an independent medical specialty in some countries. Therefore, the number of vascular professionals worldwide can only be estimated with approximation. Our sample is the largest one compared to all other relevant studies, but the degree it represents the global vascular society is only an estimation.

## Ethics Statement

The survey was approved by the ethics committee of the institution of the primary investigator. The European Union's GDPR was taken into consideration in preparing all materials of this research. No funding was received. Anonymized data are archived both offline and online at http://dx.doi.org/10.17632/fv5ztss3yf.2.

## CRediT Author Statement

**N. Patelis**: Concept, design, data collection, data analysis, writing, critical appraisal of the manuscript; **T. Bisdas**: Design, data analysis, critical appraisal of the manuscript; **Z. Jing**: Data collection, data analysis, critical appraisal of the manuscript; **J. Feng**: Data collection, data analysis, critical appraisal of the manuscript; **M. Trenner**: Data collection, data analysis, writing, critical appraisal of the manuscript; **N. Nugroho**: Data collection, data analysis, critical appraisal of the manuscript; **P.E. Reis**: Data collection, data analysis, critical appraisal of the manuscript; **S. Elkouri**: Data collection, data analysis, critical appraisal of the manuscript; **A. Lecis**: Data collection, data analysis, critical appraisal of the manuscript; **L. Karam**: Data collection, data analysis, critical appraisal of the manuscript; **D. Le Roux**: Data collection, data analysis, critical appraisal of the manuscript; **M. Ionac**: Data collection, data analysis, critical appraisal of the manuscript; **M. Berczeli**: Data collection; **V. Jongkind**: Data analysis, writing, critical appraisal of the manuscript; **K.K. Yeung**: Design, data collection, critical appraisal of the manuscript; **A. Katsargyris**: Data collection, critical appraisal of the manuscript; **E. Avgerinos**: Data collection, writing, critical appraisal of the manuscript; **D. Moris**: Data collection, writing, critical appraisal of the manuscript; **A. Choong**: Data collection, data analysis, critical appraisal of the manuscript; **J.J. Ng:** Data collection, data analysis, critical appraisal of the manuscript; **I. Cvjetko**: Data collection, critical appraisal of the manuscript; **G. Antoniou**: Data collection, writing, critical appraisal of the manuscript; **P. Ghibu**: Data collection, critical appraisal of the manuscript; **A. Svetlikov:** Data collection, writing, critical appraisal of the manuscript; **F. Gallardo Pedrajas**: Data collection, critical appraisal of the manuscript; **H. Ebben:** Data collection, critical appraisal of the manuscript; **H. Stepak**: Data collection, critical appraisal of the manuscript; **A. Chornuy**: Data collection, critical appraisal of the manuscript; **S. Kostiv:** Data collection, critical appraisal of the manuscript; **S. Ancetti:** Data collection, critical appraisal of the manuscript; **N. Tadayon:** Data collection, critical appraisal of the manuscript; **A. Mekkar:** Data collection, critical appraisal of the manuscript; **L. Magnitskiy**: Data collection, critical appraisal of the manuscript; **L. Fidalgo-Domingos**: Data collection, critical appraisal of the manuscript; **S. Matheiken**: Data collection, critical appraisal of the manuscript; **E. Sarutte Rosello**: Data collection, critical appraisal of the manuscript; **A. Isik**: Data collection, critical appraisal of the manuscript; **G. Kirkilesis**: Data collection, critical appraisal of the manuscript; **K. Kakavia**: Data collection, critical appraisal of the manuscript; **S. Georgopoulos**: Data analysis, critical appraisal of the manuscript.

## Declaration of Competing Interest

Nikolaos Patelis and Sean Matheiken are co-founders of the Med-PIE group (med-pie.com)

Otherwise, the authors declare that they have no known competing financial interests or personal relationships which have, or could be perceived to have, influenced the work reported in this article.

No funding was received.

## Appendix Supplementary materials

Image, application 1

Image, application 2
