# Supplementary material for: Dataset of the vascular e-Learning during the COVID-19 pandemic (EL-COVID) survey
Source: Data Brief. 2021 Oct 1;38:107442. doi: 10.1016/j.dib.2021.107442 (PMC8484229; doi:10.1016/j.dib.2021.107442)
Supplement: Supplementary file 1 [file mmc1.pdf]

e-Learning in the time of the COVID-19 pandemic (EL-COVID)

The EL-COVID survey concerns vascular e-learning activities (aka as distance learning) during the peak of the COVID-19 pandemic.
In this survey, a vascular e-Learning activity or distance learning activity is defined as any educational or training activity that is performed exclusively online (either synchronous or asynchronous) and the main topic falls under vascular or endovascular disciplines.
All data is anonymised and used only for research. Data will not be shared with third parties.
For more information, please visit the official page of the EL-COVID survey at http://med-pie.com/vascular-e-learning-during-the-covid-19-pandemic-el-covid-survey

* Required

What country do you practice in? *

Your answer

What is your gender? *

- Female
- Male
- Non-binary or LGBTQ+

What is your current position? *

- Vascular surgeon with >5yrs of practice
- Vascular surgeon with <5yrs of practice / Fellow
- Vascular trainee

How many e-Learning activities have you attended during the COVID-19 pandemic? (approximately)

- ○ 0
- ○ 1
- ○ 2-4
- ○ >4

Did you attend any NATIONAL e-learning activity? *

- ○ Yes
- ○ No

Did this national activity lead to an official accreditation (e.g. CME points or a certificate)?

- ○ Yes
- ○ No

Did you attend any INTERNATIONAL e-learning activity? *

- ○ Yes
- ○ No

Did this international activity lead to an official accreditation (e.g. CME points or a certificate)?

- ○ Yes
- ○ No

How did you choose what e-learning activity to attend? *

- ○ Based on the reputation of the institution organizing the activity
- ○ Based on the reputation of the presenter/panel
- ○ Interested in receiving official accreditation (e.g. CME points)
- ○ Interested in the activity topic
- ○ Other:

How did you find the activities that you have finally attended? *

- ○ Through direct contact from national/international society
- ○ Social media
- ○ Word of mouth
- ○ Online educational platforms
- ○ Actively searched for activities from well-known institutions
- ○ Other:

Does your employer support you in attending an e-learning activity through protected time / allocated time during working hours? *

- ○ Yes
- ○ No

What was reason for NOT attending an e-learning activity that you were interested in? *

- ○ Lack of time due to increased workload
- ○ Technical inability (e.g. slow internet connection)
- ○ Inability to isolate (e.g. crowded home environment during lock-down or busy office)
- ○ Time-zone difference
- ○ No accreditation
- ○ No interaction
- ○ More than one educational activities at the same time
- ○ Other:

What was your overall impression of e-learning activities during the COVID-19 pandemic? *

- ○ Very positive, e-learning activities could replace traditional activities in some subjects
- ○ Positive, but there is room for improvements
- ○ Neutral
- ○ Negative, most activities are hastily prepared
- ○ Very negative, e-learning activities cannot replace traditional activities in some subjects

Once you have attended a very interesting activity, is it easy to retrieve it in order to watch it again? *

- ○ Very easy (e.g. easily accessible through the original link)
- ○ Easy (e.g. on-demand content after free registration)
- ○ Hard (e.g. paid content)
- ○ Very hard (e.g. request content from institution)
- ○ Impossible (e.g. not available anymore)

Supposing very interesting data has been presented online through an e-learning activity. Is it easy to cite this presentation in a manuscript or a presentation of your own? *

- ○ Easy (the content is properly archived and there is a permanent link to it)
- ○ Hard (the content is not archived in a proper manner, e.g. without a permanent link)
- ○ I would not usually cite a presentation as a source

Would you list the e-learning activities you have attended in your CV/Resume as CME activities? *

- ◯ Definitely yes
- ◯ Some of them (e.g. those with CME points or certification)
- ◯ Definitely no

What would you improve regarding the e-learning activities?

Your answer

Please write your email address (optional). We need it for data validation, but it will not be shared with third parties.

Your answer

Submit

Never submit passwords through Google Forms.

This content is neither created nor endorsed by Google. Report Abuse - Terms of Service - Privacy Policy

Google Forms
