# Supplementary material for: Dataset of the vascular e-Learning during the COVID-19 pandemic (EL-COVID) survey
Source: Data Brief. 2021 Oct 1;38:107442. doi: 10.1016/j.dib.2021.107442 (PMC8484229; doi:10.1016/j.dib.2021.107442)
Supplement: Supplementary file 2 [file mmc2.pdf]

e-Learning in the time of the COVID-19 pandemic (EL-COVID)

1. e-Learning in the time of the COVID-19 pandemic (EL-COVID) in 中国

This survey is only for Vascular Surgeons & trainees in mainland CHINA (中国)

* 表示必填项

1 What is your gender? 您的性别是? *

- ◯ Female - 女
- ◯ Male - 男
- ◯ Other - 其他

2 What is your current position? 您目前的职位年资是什么? *

- ◯ Vascular surgeon with more than 5yrs of practice - 执业超过5年的血管外科医师
- ◯ Vascular surgeon with less than 5yrs of practice / Fellow - 执业<5年的血管外科医师
- ◯ Vascular trainee - 血管外科规培或专培生

3 How many e-Learning activities have you attended between December 15th and April 15th, 2020? (approximately) 在2019年12月15日至2020年4月15日之间，您参加了多少次网络在线学习活动? （大约） *

- ◯ 0
- ◯ 1
- ◯ 2-4
- ◯ >4

4 Did you attend any NATIONAL e-learning activity? 您参加过任何国家级的网络在线学习或网上学术交流活动吗? *

- ◯ Yes
- ◯ No

5 Did this national activity lead to an official accreditation (e.g. CME points or a certificate)? 这项国家级网络学习活动是否提供官方认证（例如CME学分或证书）？

- ◯ Yes
- ◯ No

6 Did you attend any INTERNATIONAL e-learning activity? 您参加过任何国际网络在线学习或网上学术交流活动吗? *

- ◯ Yes
- ◯ No

7 Did this international activity lead to an official accreditation (e.g. CME points or a certificate)? 该国际网络在线学习活动是否提供官方认证（例如CME学分或证书）？ *

- ◯ Yes
- ◯ No

8 How did you choose what e-learning activity to attend? (check what applies best) 您如何选择参加哪些网络在线学习活动? （请选择最佳项） *

- ◯ Based on the reputation of the institution organizing the activity - 基于活动组织机构的声誉
- ◯ Based on the reputation of the presenter/panel - 基于演讲者/小组的声誉
- ◯ Interested in receiving official accreditation (e.g. CME points) - 对官方认证感兴趣（例如CME学分）
- ◯ Interested in the activity topic - 对活动主题感兴趣
- ◯ Other - 其他

Comment

9 How did you find the activities that you have finally attended? (check what applies best) 您是如何找到最终参加的活动? （请选择最佳选项） *

- ◯ Through direct contact from national/international society - 通过国家/国际学术社团的官方直连社交媒体
- ◯ Social media - 社交媒体
- ◯ Word of mouth - 口口相传
- ◯ Online educational platforms - 在线教育平台
- ◯ Actively searched for activities from well-known institutions - 积极寻找知名中心组织的活动
- ◯ Other - 其他

Comment

10 Does your employer support you in attending an e-learning activity through protected time / allocated time during working hours? 您的上级是否支持您占用工作时间来参加网络在线学习活动? *

◯ Yes ◯ No

11 What was reason for NOT attending an e-learning activity that you were interested in? (check what applies best) 您未能参加感兴趣的网络在线学习活动的原因是什么？ （请选择最佳选项） *

◯ Lack of time due to increased workload - 由于工作量增加而缺少时间

◯ Technical inability (e.g. slow internet connection) - 技术障碍（例如互联网连接速度慢）

◯ Inability to isolate (e.g. crowded home environment during lock-down or busy office) - 环境不允许（例如繁忙的办公室或拥挤的家庭环境）

◯ Time-zone difference - 时差

◯ No accreditation - 没有认证（例如学分）

◯ No interaction - 没有互动

◯ More than one educational activities at the same time - 同时有多项网络学习活动，彼此冲突

◯ Other - 其他

Comment

12 What was your overall impression of e-learning activities during the COVID-19 pandemic? 您对COVID-19疫情期间的在线学习和学术交流活动总体印象如何? *

- ○ Very positive, e-learning activities could replace traditional activities in some subjects - 非常有益，在某些方面网络学习和交流活动可以代替传统会议形式
- ○ Positive, but there is room for improvements - 有益，但仍有改进的余地
- ○ Neutral - 中立
- ○ Negative, most activities are hastily prepared - 无益，大多数活动都是仓促而成
- ○ Very negative, e-learning activities cannot replace traditional activities in some subjects - 毫无益处，网络在线学习和学术交流活动无法替代传统会议

13 Once you have attended a very interesting activity, is it easy to retrieve it in order to watch it again? 参加了非常有意思的网络学习和在线学术交流活动，是否很容易回看相关视频? *

- ○ Very easy (e.g. easily accessible through the original link) - 非常容易（例如，可以通过原始链接轻松访问）
- ○ Example answer choice 3Easy (e.g. on-demand content after free registration) - 容易（例如，免费注册后可点播内容）
- ○ Hard (e.g. paid content) - 很难（例如，需要付费）
- ○ Very hard (e.g. request content from institution) - 非常难（例如，需要请求主办方机构）
- ○ Impossible (e.g. not available anymore) - 不能（例如，不可回看）

14 Supposing very interesting data has been presented online through an e-learning activity. Is it easy to cite this presentation in a manuscript or a presentation of your own? 假设网络在线学习或在线学术交流活动中有非常意义的数据，您是否可以很容易地在自己的论文或幻灯中引用演讲文稿? *

- ○ Easy (the content is properly archived and there is a permanent link to it) - 容易（内容已良好存档，且有永久索引号，便于引用）
- ○ Hard (the content is not archived in a proper manner, e.g. without a permanent link) - 难（内容没有良好存档，例如没有永久索引号）
- ○ I would not usually cite a presentation as a source - 我通常不引用演讲幻灯中的数据和素材

15 Would you list the e-learning activities you have attended in your *

CV/Resume as CME activities? 您是否会将您参加的在线学习活动列在您的简历中作为继续教育经历?

- Definitely yes - 当然
- Some of them (e.g. those with CME points or certification) - 会列某些（例如提供CME学分或认证的）
- Definitely no - 当然不会

16 What would you improve regarding the e-learning activities? 关于网络学习和在线学术交流活动，您有什么改进意见？（选填）

Enter answer

(optional)

17 Please share your email address (optional). We need it for data validation, but it will not be shared with third parties. 请分享您的电子邮件地址（选填），我们需要它来进行数据验证，但不会与任何第三方共享。

Enter answer

email (optional)

Powered by
SurveyLab
